# Prevalence of Side Effects of the AstraZeneca COVID-19 Vaccine: A Multicenter Experience From Pakistan

**DOI:** 10.7759/cureus.46543

**Published:** 2023-10-05

**Authors:** Taimur Haider, Javeria Ali, Syed Mushhood Ali, Aqsa Syed Iftikhar, Ahsan Ali Siddiqui, Adeeba Salahuddin Khan, Javeria Ahmed Qamar, Khadija Sohail, Adnan Anwar, Atif A Hashmi

**Affiliations:** 1 Paediatrics, District Headquarter Hospital, Jhang, PAK; 2 Internal Medicine, Abbasi Shaheed Hospital, Karachi, PAK; 3 Internal Medicine, Liaquat National Hospital and Medical College, Karachi, PAK; 4 Family Medicine, Tumair General Hospital, Riyadh, SAU; 5 Emergency Medicine, Ziauddin University, Karachi, PAK; 6 Internal Medicine, Dow University of Health Sciences, Karachi, PAK; 7 Internal Medicine, Jinnah Sindh Medical University, Karachi, PAK; 8 Physiology, Hamdard College of Medicine and Dentistry, Karachi, PAK; 9 Internal Medicine, Essa General Hospital, Karachi, PAK; 10 Pathology, Liaquat National Hospital and Medical College, Karachi, PAK

**Keywords:** pain, adverse effects, side effects, covid-19 vaccine, astrazeneca

## Abstract

Introduction

The most efficient method of combating the coronavirus disease 2019 (COVID-19) pandemic would be to use effective, safe, and proven vaccines; however, their widespread use has been hampered partly by concerns over possible adverse effects. Therefore, this study aimed to assess the prevalence of Oxford/AstraZeneca vaccine side effects among participants.

Methods

This was a multicenter, cross-sectional study conducted using a non-probability sampling technique. The duration of the study was nine months, from February 1, 2022, to October 31, 2022. The study included 900 participants who provided informed consent and had received two doses of the AstraZeneca vaccine. Demographic characteristics of participants, such as gender, age, comorbidities, AstraZeneca vaccine with both doses along with booster dose, previous exposure to COVID-19 infection, and the prevalence of any local and systemic side effects following the first and second doses of vaccine, were documented.

Results

The study findings showed that of the 900 participants, 414 (46.0%) were males and 486 (54.0%) were females; their mean age was 40.72 ± 13.47 years. Among them, 198 (22.0%) had hypertension and 144 (16.0%) had diabetes mellitus. Following the first dose of the AstraZeneca vaccine, pain at the injection site was the most commonly reported side effect in 594 (66.0%) participants. Moreover, swelling at the injection site was the most commonly reported side effect in 522 (58.0%) participants after receiving the second dose of the vaccine. The level of satisfaction showed that the majority of the 648 participants (72.0%) were satisfied with their vaccination.

Conclusion

This study concluded that pain at the injection site was the most commonly reported side effect, followed by swelling and fever after the first dose of the vaccine. Following the second dose of the vaccine, adverse effects included headache, swelling, and burning at the injection site.

## Introduction

Although vaccines save lives, there is a global issue of poor coronavirus disease 2019 (COVID-19) vaccine acceptance [1]. Fear of vaccination side effects is one factor contributing to hesitation [2]. Worldwide vaccination programs, which the World Health Organization (WHO) believes minimize 2-3 million fatalities from illnesses that can be prevented by vaccines each year [3], are not only successful from a monetary perspective but also a crucial component of preventive health care.

Because vaccines are essential for the prevention and management of infectious disease epidemics, a COVID-19 vaccine that is highly efficient, safe, well-accepted, and well-tolerated would be an invaluable tool for limiting pandemics [4]. Vaccination is one of the most cost-effective health expenditures and is successful in reaching even the most susceptible and difficult-to-reach groups [5]. A vaccine must be supported by those who are most at risk of being harmed by the illness in addition to being safe and effective [6].

The virus’s multiple genomic sequences have restricted its ability to create an efficient vaccine [7]. Because there are currently no successful vaccines, many lives have been lost. Two-hundred fifty-nine vaccine studies began on November 11, 2020. Numerous clinical studies have led to the development of several vaccines, including the COVID-19 vaccine developed by AstraZeneca [8]. In people over the age of 18, the COVID-19 vaccine by AstraZeneca is recommended for active immunization to avoid COVID-19 triggered by severe acute respiratory syndrome 2 (SARS-CoV-2). A single recombinant, replication-deficient chimpanzee adenovirus (ChAdOx1) vector containing the S glycoprotein of SARS-CoV-2 forms the monovalent vaccine developed by AstraZeneca. After administration, the S glycoprotein of SARS-CoV-2 regionally induces cellular immune reactions and neutralizing antibody responses [9].

Two separate injections of 0.5 ml each comprised the course of vaccination. A duration of four to 12 weeks after the first dose is recommended before administering the second dose. To complete the immunization course, patients who have already received the first dose of the AstraZeneca vaccine should also receive the second dose. The most commonly mentioned adverse responses were headache, lethargy, myalgia, and tiredness [10]. Injection site tenderness and pain were also common. Fatigue, generalized pain, chills, headache, and pyrexia are potential systemic adverse effects. Pain, redness, and edema are some of the side effects at the injection site [11].

After 12 days following immunization with Oxford/AstraZeneca, a study performed in the United Kingdom (UK) comparing rates of infection among a subgroup of vaccine recipients found a substantial increase in immunity [12]. The vaccine users may experience undesirable side effects from this interaction and other elements of the vaccine, such as rash, fever, headache, vertigo, joint pain, edema, pain, and redness at the site of injection [12].

Numerous studies have recommended that the AstraZeneca and Pfizer vaccines, BNT162b2 messenger ribonucleic acid (mRNA) and ChAdOx1 nCoV-19, respectively, appear to be very effective against the alpha (B.1.1.7) variant and other forms prior to the delta variant (DV) [13-16].

The term "vaccine hesitancy" describes "delay in accepting or refusing vaccines regardless of the accessibility of vaccine facilities"; it is a growing community health issue developed by false information about the efficacy and safety of vaccines [17]. This conclusion was corroborated from the perspective of COVID-19 vaccines, in which Poland’s healthcare professionals and pupils were less willing to take the vaccine due to concerns about adverse effects [18].

Although the AstraZeneca COVID-19 vaccine is frequently administered in a few nations, there is a shortage of evidence to substantiate its adverse consequences. Therefore, this study was designed to assess the experienced side effects of the AstraZeneca vaccine among participants.

## Materials and methods

This cross-sectional, multicenter study was conducted using a non-probability sampling technique. The study duration was nine months, from February 1, 2022, to October 31, 2022. Ethical approval for the study was obtained from Essa General Hospital (Essa/05/2022). The study included 900 participants who provided informed consent and had received the first and second doses of the AstraZeneca vaccine, with or without booster doses. The study excluded participants who had never received a COVID-19 immunization or who had received a vaccination with a different vaccine than that provided by AstraZeneca. In addition, severely ill patients, patients with advanced malignancies, immunodeficiency, and those on chemotherapy or radiation were not included in the study. Cases with ongoing COVID-19 infection were also excluded from the study.

We collected the participants’ information using a questionnaire. The survey participants were informed that participation was completely voluntary and that their permission was required before responding to any question. Demographic characteristics of participants, such as sexual characteristics, age, coexisting illnesses, AstraZeneca vaccine with first and second doses together with booster dose, previous exposure to COVID-19 infection, and incidence of local and general side effects after receiving the first and second doses of vaccine, were documented. The participants’ level of satisfaction was also noted.

Data were entered and analyzed using SPSS Statistics for Windows, Version 26.0 (IBM Corp., Armonk, NY, USA). Demographic details such as age, height, weight, and time period of hypertension and diabetes were documented as means and standard deviations. Frequencies and percentages are presented for demographic features (gender, comorbidities, past history of COVID-19 infection, and local and general adverse effects).

## Results

A total of 900 recipients who had gotten AstraZeneca vaccines with either the first or second dose were involved in the study. Of them, 414 (46.0%) were males and 486 (54.0%) were females, with a mean age of 40.72 ± 13.47 years. The mean weight and height of the participants were 68.0 ± 17.89 kg and 5.20 ± 0.76 feet, respectively. The mean duration of diabetes was 4.87 ± 0.78 years, and that of hypertension was 4.54 ± 2.02 years. Of the 900 participants, 198 (22.0%) had hypertension, and 144 (16.0%) had diabetes mellitus (DM). Only 72 (8.0%) of the participants had previously been exposed to the COVID-19 infection, as presented in Table 1. 

**Table 1:** The recipients’ basic demographic features (n=900) SD: standard deviation; COVID-19: coronavirus disease 2019

Variable		Mean±SD/n(%)
Age (years)		40.72±13.47
Weight (kg)		68.00±17.89
Height (ft)		5.20±0.76
Duration of hypertension (years)		4.54±2.02
Duration of diabetes mellitus (years)		4.87±0.78
Gender	Male	414(46.0%)
	Female	486(54.0%)
Hypertension	Yes	198(22.0%)
	No	702(78.0%)
Diabetes mellitus	Yes	144(16.0%)
	No	756(84.0%)
Previous COVID-19 exposure	Yes	72(8.0%)
	No	828(92.0%)

After getting the first dose of the AstraZeneca vaccine, pain at the injection site was generally reported as a side effect in 594 (66.0%) participants; after that, swelling at the injection site was reported in 432 (48.0%) and fever was reported in 414 (46.0%). Moreover, 396 (44.0%) participants reported myalgia. Furthermore, joint pain 360 (40.0%), burning at the injection site 324 (36.0%), and chills were reported by 288 (32.0%) participants. Contrarily, the least common adverse effects were nausea and cough, which were both reported by 90 (10.0%) and 90 (10.0%) participants, respectively, as presented in Table 2. 

**Table 2:** The distribution of side effects after first dosage of AstraZeneca vaccine

Variable	Yes, n(%)	No. n(%)
Pain at injection site	594(66.0%)	306(34.0%)
Swelling at injection site	432(48.0%)	468(52.0%)
Redness at injection site	144(16.0%)	756(84.0%)
Lymphadenopathy	216(24.0%)	684(76.0%)
Fever (temperature >37.8 ˚C)	414(46.0%)	486(54.0%)
Headache	108(12.0%)	792(88.0%)
Nausea	90(10.0%)	810(90.0%)
Rashes	216(24.0%)	684(76.0%)
Burning at injection site	324(36.0%)	576(64.0%)
Flu-like illness	252(28.0%)	648(72.0%)
Anxiety	180(20.0%)	720(80.0%)
Myalgia	396(44.0%)	504(56.0%)
Fatigue	216(24.0%)	684(76.0%)
Joint pain	360(40.0%)	540(60.0%)
Chills	288(32.0%)	612(68.0%)
Cough	90(10.0%)	810(90.0%)
Swelling of glands	270(30.0%)	630(70.0%)
Sore throat	270(30.0%)	630(70.0%)
Shortness of breath	162(18.0%)	738(82.0%)
Diarrhea	270(30.0%)	630(70.0%)
Chest pain	324(36.0%)	576(64.0%)

After receiving the second dosage of the AstraZeneca vaccine, swelling at the injection site was the most commonly observed side effect in 522 (58%) participants, followed by burning at the injection site 432 (48%), and headache 396 (44%). Moreover, fever (360, 40%), joint pain (342, 38%), rashes (324, 36%), chills (324, 36%), and gland swelling were reported by 306 (34%) participants. Likewise, the least reported side effect was localized redness that was noticed by 54 (6%) participants who were getting the second dose, and none of the participants reported nausea, as shown in Table 3. 

**Table 3:** The distribution of side effects after second dose of AstraZeneca vaccine

Variable	Yes, n(%)	No, n(%)
Pain at injection site	288(32.0%)	612(68.0%)
Swelling at injection site	522(58.0%)	378(42.0%)
Redness at injection site	54(6.0%)	846(94.0%)
Lymphadenopathy	288(32.0%)	612(68.0%)
Fever (temperature >37.8 ˚C)	360(40.0%)	540(60.0%)
Headache	396(44.0%)	504(56.0%)
Nausea	0(0.0%)	900(100.0%)
Rashes	324(36.0%)	576(64.0%)
Burning at injection site	432(48.0%)	468(52.0%)
Flu	162(18.0%)	738(82.0%)
Anxiety	270(30.0%)	630(70.0%)
Myalgia	270(30.0%)	630(70.0%)
Fatigue	198(22.0%)	702(78.0%)
Joint pain	342(38.0%)	558(62.0%)
Chills	324(36.0%)	576(64.0%)
Cough	126(14.0%)	774(86.0%)
Sore throat	180(20.0%)	720(80.0%)
Shortness of breath	252(28.0%)	648(72.0%)
Diarrhea	234(26.0%)	666(74.0%)
Chest pain	162(18.0%)	738(82.0%)

Comparison of the first-dose side effects of AstraZeneca revealed that pain and swelling at the injection site were significantly higher in the 50-year-old group than in the over-50-year-old group. Males experienced pain at the injection site more commonly, whereas females experienced swelling at the injection site more often. Non-hypertensive patients experienced pain at the injection site more often, whereas hypertensive patients experienced swelling at the injection site more frequently. Swelling at the injection site was significantly associated with DM, as shown in Table 4.

**Table 4:** The association of commonest side effects with respect to age groups, gender, and comorbidities after first dose of AstraZeneca vaccine *p-value significant as < 0.05

Variables		Pain at injection site			Swelling at injection site		
		Yes , n(%)	No, n(%)	p-value	Yes, n(%)	No, n(%)	p-value
Age group	<30 years	252(70.0%)	108(30.0%)	0.031*	162(45.0%)	198(55.0%)	<0.001*
	31-50 years	180(66.7%)	90(33.3%)		198(73.3%)	72(26.7%)	
	>50 years	162(60.0%)	108(40.0%)		72(26.7%)	198(73.3%)	
Gender	Male	288(69.6%)	126(30.4%)	0.037*	108(26.1%)	306(73.9%)	<0.001*
	Female	306(63.0%)	180(37.0%)		324(66.7%)	162(33.3%)	
Hypertension	Present	108(54.5%)	90(45.5%)	<0.001*	126(63.6%)	72(36.4%)	<0.001*
	Absent	486(69.2%)	216(30.8%)		306(43.6%)	396(56.4%)	
Diabetes Mellitus	Present	90(62.5%)	54(37.5%)	0.333	108(75.0%)	36(25.0%)	<0.001*
	Absent	504(67.7%)	252(33.3%)		324(42.9%)	432(57.1%)	

The association of second-dose side effects revealed a significant association between age and the most common side effects. Pain and swelling at the injection site were significantly more frequent in the middle age group (31-50 years). Swelling at the injection site was more frequent in males than in males, whereas swelling at the injection site was associated with male gender. Pain at the injection site was significantly associated with hypertension. Similarly, pain and swelling at the injection site were significantly associated with DM, as shown in Table 5.

**Table 5:** The association of commonest side effects with respect to age groups, gender, and comorbidities after second dose of AstraZeneca vaccine *p-value significant as < 0.05

Variables		Pain at injection site			Swelling at injection site		
		Yes, n(%)	No, n(%)	p-value	Yes, n(%)	No, n(%)	p-value
Age group	<30 years	54(15.0%)	306(85.0%)	<0.001*	234(65.0%)	126(35.0%)	<0.001*
	31-50 years	162(60.0%)	108(40.0%)		198(73.3%)	72(26.7%)	
	>50 years	72(26.7%)	198(73.3%)		90(33.3%)	180(66.7%)	
Gender	Male	36(8.7%)	378(91.3%)	<0.001*	270(65.2%)	144(34.8%)	<0.001*
	Female	252(51.9%)	234(48.1%)		252(51.9%)	234(48.1%)	
Hypertension	Present	90(45.5%)	108(54.5%)	<0.001*	126(63.6%)	72(36.4%)	0.069
	Absent	198(28.2%)	504(71.8%)		396(56.4%)	306(43.6%)	
Diabetes Mellitus	Present	108(75.0%)	36(25.0%)	<0.001*	108(75.0%)	36(25.0%)	<0.001*
	Absent	180(23.8%)	576(76.2%)		414(54.8%)	342(45.2%)	

**Figure 1:**
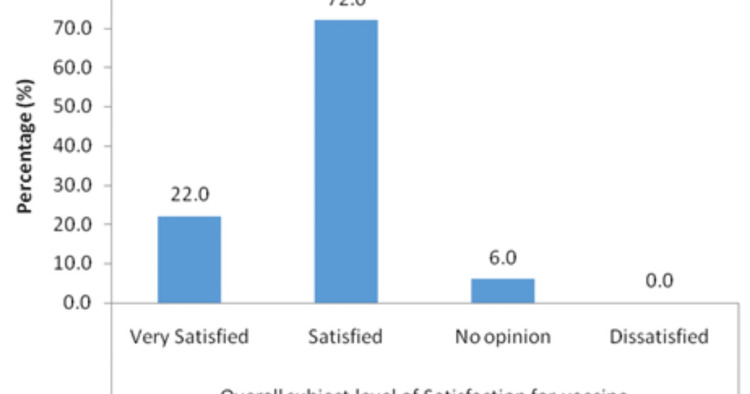
The level of satisfaction for AstraZeneca vaccine

The level of satisfaction showed that the majority of the participants, 648 (72%) were satisfied and 198 (22%) were highly satisfied with their vaccination, as presented in Figure 1.

## Discussion

The COVID-19 virus has caused illnesses and fatalities worldwide. The most efficient way to protect the world from the virus is through effective vaccination [19]. In this research, we investigated the post-vaccination adverse effects of the AstraZeneca vaccine among participants.

Similarly, one of the cross-sectional studies reported that the frequency of at least one adverse effect after receiving the first and second doses of the AstraZeneca vaccine was 91.3% and 67%, respectively. Moreover, injection site pain reported after receiving the first and second dosages was 63.8% and 50.4%, respectively. Likewise, the other reported side effects following the first and second doses of the vaccine were headaches (48.8% vs. 33.5%), fever (38.8% vs. 20.9%), myalgia (38.8% vs. 21.7%), tiredness (26% vs. 28.7%), soreness at the site (27.6% vs. 21.7%), and arthritis (27.6% vs. 20.9%) [20]. Our study was inconsistent with the above reported study and revealed that the frequently reported adverse events after receiving both doses of vaccine were pain at the site of injection (66.0% vs. 32.0%), injection site swelling (48.0% vs. 58.0%), fever (46.0% vs. 40.0%), muscle pain (44.0% vs. 30.0%), and joint pain (40.0% vs. 38.0%).

In addition, the overall results of another study showed that the vaccine’s adverse effects are generally considered mild. More than 90% of the participants reported adverse effects after receiving the first dose. None of these symptoms were severe enough to require hospitalization, and the frequency of side effects was lower (69.7%) during the course of the second dose of the vaccine than after the first dose [20]. This finding is consistent with a cross-sectional study conducted in Germany, which reported a minimum of one side effect (88.1%) [21]. A cross-sectional study of Poland revealed that the AstraZeneca vaccine’s first administered dose resulted in 96.5% of recipients reporting at least one side effect. 17.1% of the study participants expressed all adverse effects [22]. This study supported the above findings and showed that the reported side effects were mild in intensity and did not require hospitalization. In addition, after receiving the first dose of the vaccine rather than the second dose, the adverse effects were more noticeable.

Interestingly, according to another study finding, the most frequently reported symptom was injection site pain, which was reported by 63.8% and 50.4% following receiving both (first and second) dosages of the vaccine, respectively. Moreover, headaches (48.8% and 33.5%), high temperatures (38.8% and 20.9%), muscular pain (38.8% and 21.7%), tiredness (26.0% and 28.7%), injection site soreness (27.6% and 21.7%), and pain in the joints (27.6% and 20.9%) were observed after the first and second doses of the vaccine, respectively [20]. When a substance is injected into a constricted muscle, the area becomes painful. Numerous investigations have found that the most common post-vaccination adverse effect was pain at the injection site [20]. Similarly, another cross-sectional study of health workers in the Czech Republic revealed that approximately 89.8% of workers reported pain at the injection site, tiredness (62.2%), headaches (45.6%), myalgia (37.1%), and chills (33.9%) [23]. Likewise, one more study that involved residents of Saudi Arabia documented the short-term adverse effects of receiving both doses of the COVID-19 vaccine. The study found that headaches, fever, flu-like symptoms, injection site pain, and fatigue were the most prevalent symptoms [24]. These findings were partially similar to those of the present study and revealed that injection site pain was the most frequent side effect, reported by 594 (66.0%) recipients following the first dose, whereas injection site swelling was reported by 522 (58.0%) recipients after receiving the second dose. Furthermore, fever (46.0% vs. 40.0%), muscle pain (44.0% vs. 30.0%), joint pain (40.0% vs. 38.0%), and headaches (12.0% vs. 44.0%) were also observed after both vaccine doses.

Similarly, a Saudi Arabian study found that the most common adverse effects, such as general pain, tiredness, flu-like symptoms, fever, and soreness at the injection site, were not alarming [19]. These results were consistent with those of another randomized control study that noticed a decreased prevalence of serious adverse events and described comparable side effects [25]. These results agreed with those of a study conducted by Alhowaymel et al. [26] in Saudi Arabia. These findings were corroborated with the present study results and revealed that the most often reported side effects were swelling at the injection site, pain, fever, muscular and joint pain, lethargy, and headaches that were mild in intensity; whereas no serious adverse events were observed.

In addition, one study found that the least frequent side effect following COVID-19 vaccination was shortness of breath [19]. El-Shitany et al. [24] found identical results. The present study was not in agreement with the abovementioned studies and showed that following the first dosage of the vaccination, the least common side effects were nausea and cough, whereas redness at the site of injection was the least reported side effect after receiving the second dose.

Furthermore, another study found that younger individuals were more likely to experience COVID-19 vaccine side effects. Because most participants were between the ages of 18 and 25, this result was probably accurate [19]. Older people are less likely to have online access or computer abilities, which could have led to age-based sampling errors. Comparable studies, however, connect this result to the younger population’s stronger immune reaction and more prominent cytokine release [24,27]. According to El-Shitany et al. [24], people aged 60 years show flu-like symptoms. Additionally, according to Alghamdi et al. [27], individuals aged 50 years or younger had a greater frequency and severity of COVID-19 post-vaccination symptoms. As far as the present study was concerned, all the vaccinated participants were between the ages of 27 and 55 years and the majority of them experienced flu-like symptoms after receiving either of the doses.

This study was limited by the fact that it was cross-sectional and relied on participants’ reported side effects, which could have been influenced by participants’ bias and false beliefs about vaccinations. Moreover, severity of side effects was not evaluated in our study. 

## Conclusions

This study concluded that injection site pain was a frequently reported side effect, followed by swelling and fever after the first dose of the vaccine. After getting the second dose of the vaccine, the adverse effects included migraines, swelling, and burning at the site of injection. The majority of the symptoms were transient, mild, and the participants did not need to be hospitalized. Therefore, it is advised to spread initiatives to distribute correct information regarding probable adverse reactions to the Oxford/AstraZeneca vaccine, as well as to improve surveillance for adverse reactions after vaccination.
